# Editorial: Nutrition and Growth in Children With Chronic Kidney Disease

**DOI:** 10.3389/fped.2019.00402

**Published:** 2019-10-01

**Authors:** Kristen Sgambat, Sun-Young Ahn, Marva Moxey-Mims

**Affiliations:** Children's National Health System, Washington, DC, United States

**Keywords:** nutrition, growth, CKD - chronic kidney disease, protein energy wasting, appetite, IDPN, enteral, malnutrition

Proper nourishment during infancy, childhood, and adolescence is essential to support growth, development, and overall health and well-being. However, there are numerous challenges to achieving this goal in children with chronic kidney disease (CKD), as dysregulation of numerous systems impacts nutrition and growth in this population.

The Research Topic “Nutrition and Growth in Children with Chronic Kidney Disease” comprises a collection of five articles that provide an overview of nutrition and growth in the pediatric CKD population. This collection of articles span topics that include the underlying pathophysiology of disordered nutrition and growth, strategies in enteral and parenteral nutrition support, as well as an update on the growth outcomes of children with CKD in the current era.

“Determinants of Statural Growth in European Children With Chronic Kidney Disease: Findings From the Cardiovascular Comorbidity in Children With Chronic Kidney Disease (4C) Study” by Behnisch et al., reports on growth outcomes and the factors impacting growth in a large multi-center population of European children with CKD. The study found that parental height, pubertal status, and use of recombinant growth hormone were associated with improved growth in children with CKD, while syndromic diseases, later CKD stage, and higher BMI were associated with lower height standard deviation scores. Interestingly, use of recombinant growth hormone was low, with 85% of children who had a height less than the 3rd percentile not receiving growth hormone.

To provide a deeper understanding of the growth abnormalities seen in children with CKD, the article “Growth and Nutrition in Pediatric Chronic Kidney Disease” by Silverstein delves into the pathology that underlies poor linear growth in this population. The article provides a review of the multifactorial causes of dysfunctional growth affecting children with CKD, as well as current standards for nutritional assessment and treatment options to optimize growth in this population.

Protein energy wasting (PEW) is a distinct form of malnutrition that affects growth and is common among both adults and children with CKD. The intricacies of PEW in general, as well as specific aspects unique to pediatric CKD, are explored in the article “Malnutrition in Chronic Kidney Disease,” by Iorember. Key points about common micronutrient deficiencies affecting children with CKD, which may sometimes be overlooked, are also discussed.

While the ultimate goal is to optimize growth and nutrition in children with CKD, this is often challenging in clinical practice. This Research Topic therefore includes articles by experts in the respective fields of enteral and parenteral nutrition to provide guidance for the clinical implementation of these nutritional therapies. “Optimizing enteral nutrition for growth in pediatric chronic kidney disease” by Nelms, provides practical guidance and best-practice strategies to deliver optimal nutrition to infants and children through enteral nutrition support. Careful consideration is given to the special nutrient requirements of the pediatric CKD and dialysis populations, including protein, fluid, electrolytes, vitamins, and minerals. In children receiving hemodialysis, intradialytic parenteral nutrition (IDPN) is another therapeutic option to treat malnutrition. “Intradialytic Parenteral Nutrition in Pediatrics” by Juarez reviews the indications and goals of IDPN, and also provides a clinical protocol for the implementation and monitoring of this highly specialized therapy in clinical practices.

In summary, this Research Topic, “Nutrition and Growth in Children with Chronic Kidney Disease,” provides an in-depth exploration of issues affecting appetite, nutrition, growth, and development of children across the spectrum of CKD. Practical strategies for the management and treatment of nutritional issues are also addressed. This collection of articles may serve as both a knowledge resource and clinical tool kit for the nutritional management of children with CKD.

## Author Contributions

All authors listed have made a substantial, direct and intellectual contribution to the work, and approved it for publication.

### Conflict of Interest

The authors declare that the research was conducted in the absence of any commercial or financial relationships that could be construed as a potential conflict of interest.

